# Students’ Psychological Adjustment in Normative School Transitions From Kindergarten to High School: Investigating the Role of Teacher-Student Relationship Quality

**DOI:** 10.3389/fpsyg.2019.01238

**Published:** 2019-05-29

**Authors:** Claudio Longobardi, Michele Settanni, Laura Elvira Prino, Matteo Angelo Fabris, Davide Marengo

**Affiliations:** ^1^ Department of Psychology, University of Turin, Turin, Italy; ^2^ Department of Philosophy and Educational Science, University of Turin, Turin, Italy

**Keywords:** teacher-student relationship, school transition, school adjustment, primary school, middle school, high school

## Abstract

School transitions require students to adapt to new challenges and situations and can increase the risk of externalizing and internalizing psychological symptoms. The teacher-student relationship seems to be a protective factor for the risk of developing psychological symptoms during school transitions. The aim of the present study is to investigate whether the quality of the teacher-student relationship predicts the decrease of internalizing and externalizing symptoms during three school transitions, namely: from kindergarten to primary school (T1), from primary to middle school (T2), and from middle to high school (T3). We recruited 127 kindergarten students (mean age = 5.40, SD = 0.49), 113 fifth grade primary school students (mean age = 10.64, SD = 0.54), and 240 eighth grade students (mean age = 13.88, SD = 0.37) and their teachers (response rate = 95%). Data were collected from 2016 to 2018. Teachers filled out an anonymous survey, acting as informants for the students, reporting demographic details (age, gender), psychological symptoms, and quality of the teacher-student relationship. The data show that a positive teacher-student relationship quality tends to be associated with a reduction of psychological symptoms. A stable, low-conflict teacher-student relationship was confirmed as a protective factor from increased internalizing and externalizing symptoms during all normative school transitions. Furthermore, we see that an increase in teacher-student conflict during the transitions from primary to middle school, and from middle to high school is linked to an exacerbation in students’ externalizing symptoms during the first year of attendance of the new school. Our study confirms the importance of the teacher-student relationship in reducing psychological symptoms associated with school transitions, in every type of transition, favoring an improved psychological adjustment to the new environment. A positive teacher-student relationship represents a protective factor for the development of students. Study limitations are discussed.

## Introduction

During their scholastic career, students must cope with several school transitions, and each of them poses challenges on both an educational and a psychological level (Virtanen et al., 2019). Even though, for many students, the transition is a success, for some there is an observable reduction in their psychological well-being. In some studies, it is proposed that school transitions can be associated with an increment in depressive symptoms (Rueger et al., 2014), diminished self-esteem (Jindal-Snape and Miller, 2008), internalizing and externalizing symptoms, and a decrease in academic competence (Moilanen et al., 2010; Virtanen et al., 2019). Indeed, school transitions require a series of changes and adjustments to new people, environments, and needs, which may be perceived by minors as a source of stress. Transitions constitute challenges of living (Mruk, 2006), that is, situations in which individuals find themselves in a new environment, different and unfamiliar, where they experience meaningful situations and face new challenges. The passage to a new school setting requires students to face a series of transformations; the transition between kindergarten and primary school can be difficult and stressful (Quaglia et al., 2013; Wong, 2018; Wong and Power, 2019). In transitioning to first grade, children discover changes in their curriculum, with more time being dedicated to study activities and less time being devoted to play, changes in the school roles and in the relationships with the adults and their schoolmates (Wong and Power, 2019). In turn, during the transition from primary to secondary school, students face multiple changes, including moving to a new school, classroom with larger groups of students, an increase in the number of teachers, with different teaching style and expectations about their students’ behavior (Jindal-Snape and Foggie, 2008; Jindal-Snape and Miller, 2008). Furthermore, children coming from complicated family environments may have difficulties in creating successful attachment bonds (Jindal-Snape and Foggie, 2008; Gullo et al., 2015; Pellerone et al., 2017). The transition from middle to high school is the most problematic, leading to high rates of school dropout (Longobardi et al., 2016b, 2019b). During this transition, students find themselves in bigger and more chaotic environments, with a substantial increase in workload. Teachers are often perceived as cold, impersonal, and unreceptive to students’ needs, and students must restructure their friendship networks (Scalera and Alivernini, 2010; Marengo et al., 2018b).

Promoting students’ adjustment during school transitions is important for their academic outcomes (Birch and Ladd, 1997; Cappella et al., 2019), and for their psychological, emotional, and relational well-being (Longobardi et al., 2016a,b). Children who present poor scholastic adjustment face the risk of academic failure, antisocial behaviors, and becoming school dropouts (Marengo et al., 2018a). Therefore, it is important to study the factors that promote a better adjustment for students to the various transitions that they encounter in the educational system.

From a relational point of view, the existing literature recognizes an important predictive factor of students’ scholastic adjustment in the quality of the teacher-student relationship (Birch and Ladd, 1997; Pianta, 1999; Longobardi et al., 2016a,b, 2018b). From the point of view of attachment theory, teachers can contribute to the psychological world of children by offering positive relational models that are characterized by the recognition and respect of the needs of the minor, availability, and responsiveness (Pianta, 1999). The literature highlights how a positive teacher-student relationship quality, characterized by low conflict, promotes the perception of teacher support for students, and a reduction of their negative experiences (Longobardi et al., 2019a). More in general, a good quality of the relationship between teachers and students tends to promote a positive classroom environment, a higher sense of belonging and community, as well as a sense of exchange and cooperation. Therefore, it is likely that the relational experiences of students with their teachers are interiorized and proposed again with the class group (Wentzel, 2002). The evidence suggests that children who establish relationships with their teachers that are characterized by support and low conflict are more accepted by their peers and present more positive relationships with the class group (Hughes et al., 2001; Prino et al., 2016). Conflict in the relationship with the teacher tends to be positively associated with a decrease in prosocial behaviors and an increase in aggressiveness toward peers (Birch and Ladd, 1997; Wentzel, 2002; Marengo et al., 2018a), behavior problems, and a decrease of competence behaviors (Pianta et al., 1995). A conflictual relationship with teachers can increase the risk of being involved in bullying, both as a bully and as a victim (Longobardi et al., 2018a; Marengo et al., 2018a). Furthermore, children with a high level of dependence present aggressive behaviors (Howes et al., 1994), anxiety (Zee and Roorda, 2018) and difficulties in adapting to the school environment (Birch and Ladd, 1997; Longobardi et al., 2017).

A close relationship with an adult who does not belong to the family can constitute a resource for children’s development, helping them develop positive beliefs on themselves and the others, and develop adaptive social competences (Pianta, 1999). Furthermore, a positive relationship with the teacher becomes the setting in which the student can obtain support to achieve satisfactory learning (Hamre and Pianta, 2001). Therefore, a quality relationship with teachers can influence the developmental trajectory of students, fostering improved school adjustment in terms of learning processes and academic outcomes. This is important, especially for students who present emotional and behavioral difficulties, which impair both learning and the relationship with the class group, such as children with internalizing and externalizing symptoms. Both students with internalizing and externalizing problems present a lower quality relationship with their teachers (Birch and Ladd, 1998; Howes, 2000; Murray and Murray, 2004; Walker and Graham, 2019). Students with externalizing symptoms, such as impulsiveness, aggressiveness, and oppositional behavior, tend to present a low-quality relationship with teachers (Murray and Murray, 2004) that is characterized by conflict (Longobardi et al., 2016a,b), and tend to be more disruptive in the relationship with their teachers compared to their peers with internalizing symptoms (Birch and Ladd, 1998; Howes, 2000). Conversely, children with internalizing symptoms, such as anxiety and depression, tend to be less close to their teachers (Zee and Roorda, 2018). In adolescence, being more connected to teachers seems to diminish the involvement in risky behaviors (Voisin et al., 2006), and a positive teacher-student relationship promotes prosocial behaviors and responsibility (Wentzel, 2002), while incrementing psychological well-being (Reddy et al., 2003; Herrero et al., 2006).

Therefore, it seems that, globally, the teacher-student relationship offers children the support needed to acquire competences that can be useful when adapting to new environments and their connected challenges. From an evolutionary standpoint, it is legitimate to think that the teacher-student relationship plays a performing role in the development of the child in the preschool period and during the first years of school. Evidence suggests that a good-quality teacher-student relationship predicts positive scholastic outcomes and a good adjustment to the following education cycles (Hamre and Pianta, 2001). However, it seems that the quality of the teacher-student relationship continues to be considered a protective factor for a range of problem outcomes well into adolescence (Longobardi et al., 2016a,b, 2018b).

If school transitions can determine a decrease in the psychological well-being of the individual, and if the teacher-student relationship appears to be a protective factor with respect to psychological distress, it is important to analyze, in the various types of school transition, if a quality teacher-student relationship supports students in both the transition itself and in their psychological well-being. Some evidence points in this direction. For example, Silver et al. (2005) highlighted that children transitioning from kindergarten to primary school exhibited a decrease in externalizing symptoms in primary school when they had a closer relationship with their teachers, especially in children with higher levels of internalizing symptoms. O’Connor et al. (2011) emphasized that, during primary school, high-quality teacher-student relationships predicted fewer externalizing symptoms and decreased the long-term effects of internalizing symptoms. Rueger et al. (2014) instead observed that perceived teacher support reduced depressive symptoms as long as 20 months into the transition to middle school.

Despite the possible protective role of the teacher-student relationship with respect to psychological symptoms, the literature concerning school transitions has focused mostly on academic outcomes as a measure of school adjustment, and not as much on the psychological aspects of the transition. Still, motivation and commitment to learning and emotional-behavioral factors are two aspects that are connected and that influence each other. Furthermore, the majority of studies available, to our knowledge, have focused primarily on one type of transition, and this is a limitation because it decreases the possibility of considering the quality of the teacher-student relationship as a protective factor with respect to the psychological symptoms that are associated to school transitions in the various phases of development.

In light of our previous considerations, the present study investigated the role of changes in teacher-student relationship quality as a factor in students’ psychological adjustment during the normative school transitions from kindergarten to primary school, from primary to middle school, and from middle to high school. In particular, we examined the role of transition-related changes in teacher-student conflict and closeness as potential predictors of an increase in internalizing and externalizing symptoms during school transitions. Before presenting the methods and data of our research, we believe that a brief digression on the transitions expected by the Italian school system might be useful to help readers from other nations better understand the nature of our study. Formal schooling begins at age 6, but children can access kindergarten at age 3. The first transition concerns the passage from kindergarten to primary school. The latter lasts 5 years; subsequently, students move onto the second school cycle, that is, middle school, which lasts 3 years. Afterward, following a state exam, students can choose freely among a series of high schools, which last 3–5 years depending on the curriculum, and which are concluded with another state exam. In each of the cycles, students are linked to the didactic structures and remain with the same class group. Schooling is mandatory up to age 16.

## Materials and Methods

### Participants and Procedure

We invited 483 students and their teachers for participation in the research. Convenience sampling was used. Criteria for students’ inclusion were (1) signed informed consent by students’ parents and teachers and (2) expected transition to the next school level during the following year.

Eventually, 24 (5%) students and their parents did not agree to participate. Thus, the initial sample consisted of 459 kindergarten, primary, and middle school students and their teachers from Northern Italy. Specifically, 127 participants consisted of kindergarten students who were expected to transition to primary school (fifth grade) later in the year; 113 participants were (fifth grade) primary school students expected to transition to middle school (*N* = 113); 240 were (eighth grade) middle school students transitioning to high school. In the present study, teachers acted as informants for students, providing information regarding students’ demographic characteristics (age, gender), quality of teacher-student relationship, and students’ psychological symptoms. We collected data using a longitudinal design including a baseline assessment at the students’ current school (school year 2016–2017) and a 1-year follow-up assessment in the new school; 141 participants were lost at follow-up given that some of the new schools did not give consent for the research to continue. The final sample consists of 308 students, namely 104 kindergarten students (49% female; mean age = 5.40, SD = 0.49), 97 primary school students (46% female; mean age = 10.64, SD = 0.54), and 107 middle school students (50% female; mean age = 13.88, SD = 0.37).

### Ethical Considerations

School principals gave their consent for the participation of both teachers and students in our study. Individual informed consent to take part in the research was also collected from teachers, children, and their parents, along with written consent describing the nature and objective of the study according to the ethical code of the Italian Association for Psychology (AIP). The consent stated that data confidentiality would be assured, and that participation was voluntary. For the pupils, both parents were asked to sign the consent form in order to have their child participate in our study. The study was approved by the IRB of the University of Turin (approval number: 42345).

### Instruments

#### Psychological Symptoms

Teachers provided a rating of students’ symptoms by answering the teacher version of the Italian version of the Strength and Difficulties Questionnaire [SDQ; (Goodman, 1997; Tobia and Marzocchi, 2011)], which includes 25 items that refer to the positive or negative traits of a student’s behavior in class. The items are evaluated on a 3-point Likert scale (i.e., *Not True, Partially True, Absolutely True*), and assess five dimensions of children’s emotional and behavior characteristics: Emotional problems (e.g., “Often unhappy, downhearted”), Conduct problems (e.g., “Often fights with other children”), Hyperactivity/Inattention (e.g., “Easily distracted, concentration wanders”), Peer relationship problems (e.g., “Has at least one good friend,” reversed), and Prosocial behavior (e.g., “Considerate of other people’s feelings”). As suggested by Goodman et al. (2010), items for the emotional symptoms and problematic relationships with peers were combined to compute a score for internalizing symptoms (kindergarten to primary school: *α*_T1_ = 0.70 to *α*_T2_ = 0.83; primary to middle school: *α*_T1_ = 0.64 to *α*_T2_ = 0.75; middle to high school: *α*_T1_ = 0.78 to *α*_T2_ = 0.63), while conduct problems and hyperactivity and inattention symptoms were combined to compute a score for externalizing symptoms (kindergarten to primary school: *α*_T1_ = 0.86 to *α*_T2_ = 0.92; primary to middle school: *α*_T1_ = 0.85 to *α*_T2_ = 0.88; middle to high school: *α*_T1_ = 0.86 to *α*_T2_ = 0.75).

#### Teacher-Student Relationship Scale

The Teacher-student Relationship Scale (STRS) is a self-report instrument consisting of 28 items developed with reference to Attachment Theory (Pianta, 2001). The items are evaluated on a 5-point Likert scale. The final form of the scale presents three factors, identified as the Conflict, Closeness, and Dependency subscales. The original instrument by Pianta has been adapted and validated to the Italian context (Fraire et al., 2013; Settanni et al., 2015). For the purpose of this study, we administered the subscale for Closeness (kindergarten to primary school: *α*_T1_ = 0.91 to *α*_T2_ = 0.95; primary to middle school: *α*_T1_ = 0.85 to *α*_T2_ = 0.82; middle to high school: *α*_T1_ = 0.87 to *α*_T2_ = 0.81) and Conflict (kindergarten to primary school: *α*_T1_ = 0.86 to *α*_T2_ = 0.90; primary to middle school: *α*_T1_ = 0.85 to *α*_T2_ = 0.85; middle to high school: *α*_T1_ = 0.92 to *α*_T2_ = 0.73).

### Data Analysis

As a first step, for each time point of the considered normative school transitions, we computed descriptive statistics (i.e., mean, standard deviation) for the study variables. As a next step, we performed paired-samples *t*-tests to investigate the significance of longitudinal changes in teacher-student relationship quality and students’ psychological symptoms during students’ transition the next school level.

In order to investigate the role of teacher-student closeness and conflict in influencing students’ internalizing and externalizing symptoms, we performed a set of multiple linear regression analyses. With these models, we investigated both the impact of overall teacher-student relationship quality during the school transition, as well as changes in teacher-student relationship quality across the two time points, on students’ internalizing and externalizing symptoms after transitioning to the new school (Labouvie et al., 1991). Overall teacher-student relationship closeness and conflict are operationalized as the average of the scores before (T1) and after (T2) transitioning to the next school level, while changes are operationalized as the difference of these two scores, so that the T1 score is subtracted from the T2 score. We chose to use this analytic approach in order to examine both the effect of having stably low and high values in in teacher-student relationship quality, as well as the change in teacher-student relationship quality during the school transition. We controlled for potential collinearity among these predictors by mean centering the variables (Aiken et al., 1991). Further, in order to determine for potential multicollinearity in the predictors set, before running each model, we examined tolerance and variance inflation factor (VIF).

For each normative school transition (i.e., kindergarten to primary school, primary to middle school, middle to high school), we performed 2 regression models, one for each of the symptom variables at T2. Hence, we performed a total of six regression models; in all models, we controlled for children’s gender and symptom scores at T1. All analyses were performed using SPSS, version 18.

## Results

### Changes in Teacher-Student Relationship Quality and Psychological Symptoms During School Transitions

Table 1 shows the results of paired sample *t*-tests testing the presence of significant differences in teacher-student conflict and closeness, and students’ internalizing and externalizing symptoms, before and after transitioning to the next school level. Concerning the transition from kindergarten to primary school, we found a significant average decrease in teacher-student conflict, while no other significant difference emerged. Concerning the transition from primary to middle school, results show a significant decrease in teacher-student closeness, and a significant increase in internalizing symptoms, after transitioning to the new school. Finally, concerning the transition from middle school to high school, we found a significant decrease in teacher-student conflict, while no other significant differences emerged.

**Table 1:** Descriptive statistics and paired samples *t*-tests for student-teacher relationship and student psychological symptoms during normative school transitions.

	STRS Conflict		STRS Closeness		Internalizing Symptoms		Externalizing Symptoms	
School transition	T1 Mean (SD)	T2 Mean (SD)	T1 Mean (SD)	T2 Mean (SD)	T1 Mean (SD)	T2 Mean (SD)	T1 Mean (SD)	T2 Mean (SD)
Kindergarten to primary school	1.43 (0.68)	1.22 (0.56)^*^	4.11 (0.81)	3.98 (0.95)	0.30 (0.29)	0.28 (0.34)	0.36 (0.40)	0.37 (0.47)
Primary school to middle school	1.20 (0.63)	1.31 (0.50)	4.23 (0.38)	3.42 (0.60)^**^	0.17 (0.20)	0.25 (0.26)^**^	0.27 (0.35)	0.27 (0.37)
Middle school to high school	1.63 (0.79)	1.38 (0.48)^**^	3.40 (0.73)	3.34 (0.69)	0.33 (0.3)	0.28 (0.26)	0.46 (0.37)	0.39 (0.35)

STRS, Student-teacher relationship Scale.

* p < 0.05;

** p < 0.01.

### Teacher-Student Relationship Quality As a Predictor of Student Psychological Symptoms in Normative School Transitions

Tables 2–7 show the results of regression analyses investigating the role of teacher-student conflict and closeness as predictors of students’ internalizing and externalizing symptoms during three different school transitions, namely kindergarten to primary school, primary school to middle school, and middle school to high school. Inspection of tolerance and VIF values indicated that no problems with multicollinearity existed in the predictors set (tolerance > 0.47 and VIF < 2.12 for all predictors).

**Table 2:** From kindergarten to primary school: T2 externalizing symptoms on student-teacher relationship scale variables (*N* = 104; *R*^2^ = 0.46).

	*B*	SE	β	*t*	*p*
Intercept	0.24	0.13	–	1.83	0.07
Closeness (mean)	−0.12	0.06	−0.18	−1.97	0.05
Closeness (change)	−0.02	0.04	−0.04	−0.51	0.61
Conflict (mean)	0.25	0.09	0.30	2.85	0.01
Conflict (change)	0.09	0.07	0.11	1.35	0.18
Externalizing symptoms (T1)	0.43	0.11	0.37	3.91	<0.01
Gender (female = 1; male = 0)	−0.01	0.07	−0.01	−0.15	0.88

**Table 3:** From kindergarten to primary school: T2 internalizing symptoms on student-teacher relationship scale variables (*N* = 104, *R*^2^ = 0.30).

	*B*	SE	β	*t*	*p*
Intercept	0.14	0.10	–	1.35	0.18
Closeness (mean)	0.00	0.05	−0.01	−0.09	0.93
Closeness (change)	−0.02	0.03	−0.06	−0.68	0.50
Conflict (mean)	0.20	0.07	0.32	2.98	<0.01
Conflict (change)	−0.02	0.05	−0.04	−0.44	0.66
Internalizing symptoms (T1)	0.40	0.11	0.34	3.66	<0.01
Gender (female = 1; male = 0)	0.02	0.06	0.02	0.25	0.80

**Table 4:** From primary to middle school: T2 externalizing symptoms on student-teacher relationship scale variables (*N* = 97; *R*^2^ = 0.63).

	*B*	SE	β	*t*	*p*
Intercept	0.24	0.09	–	2.77	0.01
Closeness (mean)	0.08	0.07	0.08	1.05	0.29
Closeness (change)	−0.03	0.05	−0.05	−0.57	0.57
Conflict (mean)	0.22	0.09	0.22	2.43	0.02
Conflict (change)	0.14	0.05	0.21	2.89	<0.01
Externalizing symptoms (T1)	0.61	0.09	0.57	6.49	<0.01
Gender (female = 1; male = 0)	−0.09	0.05	−0.12	−1.69	0.09

**Table 5:** From primary to middle school: T2 internalizing symptoms on student-teacher relationship scale variables (*N* = 97, *R*^2^ = 0.26).

	*B*	SE	β	*t*	*p*
Intercept	0.17	0.08	–	2.10	0.04
Closeness (mean)	−0.01	0.07	−0.02	−0.18	0.86
Closeness (change)	0.07	0.05	0.18	1.56	0.12
Conflict (mean)	0.30	0.07	0.43	4.05	<0.01
Conflict (change)	0.05	0.05	0.12	1.16	0.25
Internalizing symptoms (T1)	0.20	0.12	0.15	1.66	0.10
Gender (female = 1; male = 0)	0.03	0.05	0.06	0.63	0.53

**Table 6:** From middle to high school: T2 externalizing symptoms on student-teacher relationship scale variables (*N* = 117; *R*^2^ = 0.39).

	*B*	SE	β	*t*	*p*
Intercept	0.35	0.10	–	3.49	<0.01
Closeness (mean)	−0.02	0.05	−0.04	−0.42	0.68
Closeness (change)	−0.10	0.03	−0.26	−2.96	<0.01
Conflict (mean)	0.25	0.07	0.35	3.66	<0.01
Conflict (change)	0.12	0.04	0.30	3.22	<0.01
Externalizing symptoms (T1)	0.37	0.08	0.39	4.45	<0.01
Gender (female = 1; male = 0)	−0.08	0.06	−0.12	−1.53	0.13

**Table 7:** From middle to high school: T2 internalizing symptoms on student-teacher relationship scale variables (*N* = 117, *R*^2^ = 0.07).

	*B*	SE	β	t	*p*
Intercept	0.21	0.09	–	2.43	0.02
Closeness (mean)	−0.03	0.05	−0.06	−0.58	0.56
Closeness (change)	−0.02	0.03	−0.07	−0.66	0.51
Conflict (mean)	0.12	0.06	0.24	2.04	0.04
Conflict (change)	0.01	0.03	0.05	0.41	0.68
Internalizing symptoms (T1)	0.10	0.08	0.12	1.30	0.20
Gender (female = 1; male = 0)	0.03	0.05	0.05	0.53	0.60

Concerning the transition from kindergarten to primary school (Tables 2 and 3), we see that controlling for children’s symptoms at T1 (kindergarten), the average of teacher-student conflict during the transition (mean of T1, T2 scores) emerges as positive predictor for children’s externalizing symptoms (*β* = 0.30, *p* = 0.01) and internalizing symptoms (*β* = 0.32, *p* = 0.01) after the transition (T2). In turn, the average of teacher-student closeness during the transition (mean of T1, T2 scores) emerges as a negative predictor of student externalizing symptoms (*β* = 0.18, *p* = 0.05) as reported after the transition to primary school (T2), while no significant effect emerges concerning children’s internalizing symptoms.

As regards the transition from primary to middle school (Tables 4 and 5), results show that both the average of teacher-student conflict during the transition (mean of T1, T2 scores) (*β* = 0.22, *p* = 0.02), and the change between the T2 and T1 conflict scores (*β* = 0.21, *p* < 0.01), emerge as positive predictors of children’ externalizing symptoms after transitioning to middle school (T2). In turn, the average of teacher-student conflict during the transition (mean of T1, T2 scores) emerges as a positive predictor of students’ internalizing symptoms (*β* = 0.43, *p* < 0.01) at T2, while the change between scores showed no significant effect.

Concerning the transition from middle to high school (Tables 6 and 7), and similar to the transition from primary to middle school, results show that both the average of teacher-student conflict during the transition (mean of T1, T2 scores) (*β* = 0.35, *p* < 0.01) and the change between the T2 and T1 conflict scores (*β* = 0.31, *p* < 0.01) emerge as positive predictors of adolescents’ externalizing symptoms during the first year of high school (T2). In turn, a positive change between the T2 and T1 closeness scores (*β* = 0.31, *p* < 0.01) is associated with a decrease in externalizing symptoms at T2 (*β* = −0.26, *p* < 0.01). Finally, the average of teacher-student conflict during the transition (mean of T1, T2 scores) emerges as a positive predictor of adolescents’ internalizing symptoms as assessed during the first of year of high school (T2).

## Discussion

The aim of the present study was to investigate the association between teacher-student relationship quality and children and adolescents’ psychological adjustment during three normative school transitions, namely the transitions from kindergarten to primary school, from primary to middle school, and from middle to high school. Several studies have confirmed that a positive teacher-student relationship is a protective factor for students’ adjustment to the school environment (Birch and Ladd, 1998; Howes, 2000; Murray and Murray, 2004; Silver et al., 2005; Rueger et al., 2014; Longobardi et al., 2016a); however, the majority of studies have mainly focused on academic outcomes and, to our knowledge, these studies have focused on a single transition.

Overall results highlight the importance of stable, low-conflict teacher-student relationships as a protective factor from increased internalizing and externalizing symptoms during all normative school transitions. Furthermore, we see that an increase in teacher-student conflict during the transitions from primary to middle school and from middle to high school is linked to an exacerbation in students’ externalizing symptoms during the first year of attendance of the new school. School transitions require children to adapt to a new school environment and to face new academic and developmental challenges (Jindal-Snape and Foggie, 2008; Longobardi et al., 2016b; Wong and Power, 2019). These requirements on behalf of the school environment can have a negative influence on students’ psychological well-being. However, it is possible that a positive quality of the teacher-student relationship might act as a protective factor for the development of internalizing and externalizing symptoms (Birch and Ladd, 1998; Howes, 2000; Murray and Murray, 2004). Our data seem to confirm this protective role of teacher-student relationships in each school transition examined. In particular, findings indicate that the influence of teacher-student relationship on children’s psychological well-being is not limited to kindergarten and primary school, when children are more dependent from the adults, but also plays an important role for adolescents’ adjustment. According to the attachment theory framework, it is possible that teachers influence the developmental trajectory of students by contributing to their psychological world and offering positive relational models, characterized by closeness, responsiveness, and recognition of their emotional needs. Therefore, it is a relationship that becomes the relational setting in which children confront themselves with a significant adult and obtain support for the challenges they encounter (Hamre and Pianta, 2001), such as those imposed by school transitions. The quality of said relationship could be vital for those children that present internalizing and externalizing symptoms, considering the difficulties that children with such symptoms encounter in their academic functioning. A positive teacher-student relationship has been shown to be associated with a decrease in internalizing and externalizing symptoms during school transitions (Silver et al., 2005; Rueger et al., 2014), predicts better school adjustment (Pianta, 1999) and a decrease in aggressive behaviors (Birch and Ladd, 1997; Wentzel, 2002; Marengo et al., 2018a), and, more in general, an improvement in the classroom environment (Pianta et al., 1995; Pianta, 1999). Therefore, it is likely that a teacher-student relationship characterized by low conflict and high closeness can provide children with the relational instruments and support needed to adjust to the new school context, and ultimately promote their psychological well-being.

If the teacher-student relationship is recognized as a protective factor of the psychological adjustment of the minor to the school environment following a transition, it is important to develop prevention and intervention strategies, able to intercept risk situations and promote more adaptive forms of relationships between students and teachers. This care should be applied to all school levels, recognizing teacher-student relationships as a protective factor with respect to psychological adjustment. Considering the influence that internalizing and externalizing symptoms have on students’ developmental trajectories, the teacher-student relationship becomes a protective factor that helps support the more general positive development of individuals (O’Connor et al., 2011).

Results from the present study have implications for research and the clinical setting. By employing a longitudinal approach, our study shows that both stability and change in quality of teacher-student relationships play a role in promoting student’s adjustment during normative school transitions, in particular as children approach adolescence. Thus, studies investigating the predictors of students’ well-being and behavioral adjustment in the period after a school transition should consider collecting information about both students’ current and past relationship with teachers. As regards clinical implications, findings from the present study provide information which can be useful for school psychologists when planning interventions aimed at improving students’ adjustment to new school contexts, as well as at identifying students at risk for behavioral problems, which in turn may increase their risk of developing problematic school behaviors (e.g., absenteeism, school refusal, truancy; Reissner et al., 2019).

### Limitations

Our study suffers from some limitations. First, we employed a convenience sampling approach, which may have compromised our ability to generalize results to the reference populations of students. Second, the small size of the recruited sample, and the presence of significant sample attrition across the time points of the study. Use of a larger, representative sample of students would have increased our ability to detect even small effects and increased the robustness of results. Third, despite collecting longitudinal data for three normative school transitions, we could only follow students over time during a single school transition, i.e., the three analyzed datasets are independent. Collecting data on the same group of students over two or more school transitions, as well as increasing the number of assessment time points during the considered school years, would have allowed us to identify both linear and nonlinear trends of teacher-student relationship quality over time and their association with students’ psychological adjustment over their life course from childhood to adolescence. Increasing the time range and assessment time points would also have allowed to put to test alternative causal relationships between the investigated constructs, e.g., by modeling the relationship between teacher-student relationship quality and student psychological adjustment using cross-lagged longitudinal models. Another limitation relates to the lack of collected information about students’ attitudes toward the school transition, e.g., students’ expectations hopes about their future transition, differences in the perception of future events are expected to significantly influence individuals’ adjustment to a new context, or status (Tsuzuki, 2012; Mannino et al., 2017). Future studies investigating students’ longitudinal adjustment during normative school transitions should consider investigating students’ time perspective as a factor in their level of adjustment to the new context. Finally, in the present study students’ teacher acted as the sole informant on students’ symptoms and teacher-student relationship quality, possibly introducing alterations in the associations between the investigated constructs due to common method bias. Further, students’ information was provided by different teachers at each time point, but we were not able to control in the analyses for the influence of teachers’ characteristics (e.g., job experience, demographic features, well-being, and health conditions) which may affect their appraisal of students’ symptoms (Fiorilli et al., 2017). In order to increase the robustness of results, future studies should consider collecting information from multiple sources, e.g., by collecting information from students’ parents or, when possible, directly from students.

## Ethics Statement

School principals gave their consent for the participation of both teachers and students in our study. Individual informed consent to take part in the research was also collected from teachers, children and their parents, along with written consent describing the nature and objective of the study according to the ethical code of the Italian Association for Psychology (AIP). The consent stated that data confidentiality would be assured, and that participation was voluntary. For the pupils, both parents were asked to sign the consent form in order to have their child participate in our study. The study was approved by the IRB of the University of Turin (approval number: 42345).

## Author Contributions

CL, LP, and MF were involved with the design and interpretation of this work as well as writing the manuscript. LP and MF were involved in the acquisition of the data. MS and DM analyzed the data and contributed to the writing of the manuscript.

### Conflict of Interest Statement

The authors declare that the research was conducted in the absence of any commercial or financial relationships that could be construed as a potential conflict of interest.
